# The Financial Side

**Published:** 1920-11-06

**Authors:** 


### A STATE NURSING SERVICE.

### The Financial Side.

Although the key to the problem is finance, yet it is not of vital importance to decide precisely how the funds for a State Nursing Service should be raised before coming to a definite conclusion on the question of principle. It is more than a matter of expediency that adequate provision should be made by the nation for the upkeep of a civilian nursing army. If the means are not forthcoming from some quarter, the process of degeneration which has already set in will become accelerated. There is no denying the fact that a decision must be arrived at without delay as to whether nursing is to be kept at a high or a low level—whether recruits are to be sought for from amongst the more enlightened and refined classes or from a class, well-meaning and honest no doubt, but uneducated and rough. Just now both sources are being tapped. The larger Metropolitan institutions are still able to maintain a satisfactory supply, but the matrons of excellent provincial hospitals bitterly complain of an increasing number of applicants who belong to the category *vulgaris*, and a diminishing number from homes where the atmosphere breathes culture and refinement.

### Charity and Service.

The root cause is economic, and the natural consequence of an endeavour to make the nurse an integral part of hospital charitable effort. The doctor often is, but the builder, the butcher, the charwoman are not. Hospital boards do not ask the latter to give their services save at the full market price. A writer in the *Woman's Leader*, calling on college girls to train, points out with perfect accuracy that fifty years ago "women of fine character and lofty aims" joined the profession because of their strong desire to serve humanity at a time when woman's field of enterprise was strictly circumscribed. This day is dead, and I am persuaded that only a lack of perception on the part of hospital authorities causes them to continue to recruit modern institutions according to archaic methods. It is courting ridicule to ask well-educated British women to nurse the nation's sick poor in times of peace and prosperity as a tribute to altruism. A million would volunteer if it were necessary; but it is not.

In passing, may I say that there is one statement in the article mentioned which I am sorry that the writer did not more fully elaborate? "There is every indication," runs the text, "that nursing is taking its place among the professions, competing—necessarily on most unequal grounds—with a host of other professions for women. And if only to hasten its levelling-up, one would like to see a rush of college girls into the ranks." The college girl will ask one pertinent question, and it is this I should like to see touched: Does nursing offer a career, and, if so, what are the possibilities?

When the nurse is economically dissociated from the hospital in which she serves, her sun will begin to rise, and the record of the past fifty years points to the improbability of substantial improvement taking place otherwise or earlier.

### Rates or Taxes.

The necessary cost must be paid either as rates or taxes. One has to look round for examples and comparisons. Take the London police. They are paid by the ratepayers of the Metropolis, but the Home Secretary administers the Force and fixes the men's wages. It is, to all intents and purposes, a State police service, and a London nursing service paid from the rates and administered by the Minister of Health would exactly correspond. The Army Nursing Service is paid out of Imperial taxation and administered by the War Office. The hospital nurse is paid from charitable funds, and she herself is expected to contribute a part of her services towards the charity. Hence the chasm that yawns between her economic position and that of the Army nurse. Her work is by no means inferior. On the contrary, it is vastly more varied and general.

The difference to the citizen between a nursing service paid from the rates and one paid out of the Treasury would probably work out somewhat in this way. Nurses paid from the rates would be available for service only within the rating area concerned, whereas if their emoluments were provided by Parliament no particular locality could make a special claim, save on the ground of sick persons requiring attendance. If a serious epidemic occurred in the Shetlands, nurses paid out of the rates could not be sent thither save at the expense of the islanders.

### An Obsolete System.

This, of course, although a very important matter, is only a detail. Before the time is ripe for its discussion a great deal of ground must be covered. The nursing world has first to realise that the present system is obsolete, and that unless it is scrapped, and a better scheme put into operation, the profession will sink to a lower level. It is idle to hope that by making appeals to the generosity of the public the nursing service of these islands can be maintained at a high standard and supplied with suitable recruits. The modern girl will make no outcry, but will quietly refrain from responding to the call.

If this is doubted, the statement may be verified by inquiry at any woman's college. IERNE.

